# The Frequency and Clinical Implication of *ROS1* and *RET* Rearrangements in Resected Stage IIIA-N2 Non-Small Cell Lung Cancer Patients

**DOI:** 10.1371/journal.pone.0124354

**Published:** 2015-04-23

**Authors:** Sha Fu, Ying Liang, Yong-Bin Lin, Fang Wang, Ma-Yan Huang, Zi-Chen Zhang, Jing Wang, Wen-Jian Cen, Jian-Yong Shao

**Affiliations:** 1 Department of Molecular Diagnostics, Sun Yat-sen University Cancer Center, State Key Laboratory of Oncology in South China, Collaborative Innovation Center for Cancer Medicine, Guangzhou, 510060, China; 2 Department of Medical Oncology, Sun Yat-sen University Cancer Center, State Key Laboratory of Oncology in South China, Collaborative Innovation Center for Cancer Medicine, Guangzhou, 510060, China; 3 Department of Thoracic Surgery, Sun Yat-sen University Cancer Center, State Key Laboratory of Oncology in South China, Collaborative Innovation Center for Cancer Medicine, Guangzhou, 510060, China; 4 Department of Pathology, Sun Yat-sen University Cancer Center, State Key Laboratory of Oncology in South China, Collaborative Innovation Center for Cancer Medicine, Guangzhou, 510060, China; H. Lee Moffitt Cancer Center & Research Institute, UNITED STATES

## Abstract

To evaluate the frequency and clinicopathological features of *ROS1* and *RET* rearrangements in N2 node positive stage IIIA (IIIA-N2) non-small cell lung cancer (NSCLC) patients, we retrospectively screened 204 cases with a tissue microarray (TMA) panel by fluorescent in situ hybridization (FISH), and confirmed by direct sequencing and immunohistochemistry (IHC). The relationship between *ROS1* or *RET* rearrangements, clinicopathological features, and prognostic factors were analyzed in resected stage IIIA-N2 NSCLC. Of the 204 cases, 4 cases were confirmed with *ROS1* rearrangement, but no *RET* rearrangement was detected. All 4 ROS1-rearranged cases were adenocarcinomas. The predominant pathological type was acinar pattern in ROS1-rearranged tumors, except for 1 case harboring a mixture acinar and mucous tumor cells. Variants of *ROS1* rearrangement were SDC4-ROS1 (E2:E32), SDC4-ROS1 (E4:E32) and SDC4-ROS1 (E4:E34). There was no significant association between *ROS1* rearrangement and clinicopathological characteristics. In this cohort, multivariate analysis for overall survival (OS) indicated that squamous cell carcinoma and lobectomy were independent predictors of poor prognosis; R0 surgical resection and non-pleural invasion were independent predictors of good prognosis. In resected stage IIIA-N2 NSCLC patients, ROS1-rearranged cases tended to occur in younger patients with adenocarcinomas. The prognosis of resected stage IIIA-N2 is generally considered poor, but patients with *ROS1* rearrangement will benefit from the targeted therapy.

## Introduction

Non-small cell lung cancer (NSCLC) accounts for approximately 85% of all lung cancers [1]. Complete resection is the most effective treatment for patients with lung cancer, but postoperative survival remains unsatisfactory, especially for the IIIA NSCLC [2,3]. IIIA NSCLC is defined as locally advanced NSCLC, and the resection rate of which is only 14–20% [4]. These patients are offered different postoperative adjuvant treatments according to different N stages. Although various strategies have been administrated in stage IIIA-N2 NSCLC, relatively poor prognosis occurs in a large portion of these patients [5]. In fact, the 5-year survival rate after surgery for IIIA-N2 NSCLC patients is approximately 20%, and 30% patients have recurrences and metastases within five years [4,6]. Significant discrepant clinical outcomes in IIIA-N2 NSCLC patients require a novel and effective therapy.

Since oncogenic genes were identified, targeted therapy has emerged as a highly effective treatment for lung cancer patients [7]. Epidermal growth factor receptor (*EGFR*) is the best-known target gene for lung cancer, which mutates in a relatively high frequency of 20–30% and tyrosine kinase inhibitors (TKIs) are particularly effective for treating these patients [8–10]. Aside from the high-frequency target sites, target gene with low frequency can also be excellent and effective therapeutic targets [7]. For instance, anaplastic lymphoma kinase (*ALK*) rearrangement showed the fusion in approximately 3–6% of NSCLC patients and the overall response rate rose to 57% in ALK-rearranged patients [11–14]. Excitingly, the second generation TKIs like ceritinib further increased the disease control rate of ALK-rearranged patients [15–17].

However, 40% of NSCLC patients still lack an effective targeted therapy option even with large-scale genotyping efforts in target gene research [7]. It is, therefore, of great urgency to identify more oncogenic drivers in the NSCLC patients, especially for the IIIA-N2 NSCLC subpopulations with poor prognosis. Notably, the fusion in receptor tyrosine kinase c-ros oncogene 1 (*ROS1*) and ret proto-oncogene (*RET*) genes offer a distinct molecular classification of NSCLC patients and show a high potential to be candidate targets [18–24]. *ROS1* gene encodes a receptor tyrosine kinase (RTK) of the insulin receptor family that maps to chromosome 6q22 [25]. *RET* gene located on chromosome 10q11 encodes a receptor from the glial cell line-derived neurotrophic factor family (GDNF) [26–28]. Preclinical work suggests that *ROS1* and *KIF5B-RET* rearrangements are sensitive to kinase inhibitors [29]. Several targeted agents to *ROS1* and *KIF5B-RET* rearrangements have been developed. Clinical activity of cabozantinib in RET-rearranged NSCLC patients has been reported and current studies indicate that patients with *ROS1* rearrangement may benefit from crizotinib [7,30].

For patients with stage IIIA-N2 NSCLC, there is considerable controversy about optimal therapy. Study concerning the value of *ROS1* and *RET* rearrangements in targeted therapy is rare, and their relationship with clinicopathological characteristics in resected stage IIIA-N2 NSCLC remains unclear.

In this study, we selected the East-Asian homogeneous cases of resected stage IIIA-N2 NSCLC, and we measured *ROS1* and *KIF5B-RET* rearrangements by fluorescent in situ hybridization (FISH), and confirmed the result via direct sequencing and immunohistochemistry (IHC). Then, we analyzed clinicopathological characteristics and overall survival (OS). We observed patients with *ROS1* or *RET* rearrangements, which may function as valuable targets for offering a novel postoperative treatment strategy for locally advanced NSCLC.

## Methods and Materials

### Study population

In this retrospective study, 288 individuals with resected stage IIIA-N2 NSCLC were selected from Sun Yat-Sen University Cancer Center (SYSUCC) between January 1999 and December 2004. A total number of 227 NSCLC patients were enrolled according to the criteria: (1) IIIA-N2 stage cancer; (2) surgery with mediastinal lymph node dissection; (3) sufficient formalin-fixed and paraffin-embedded (FFPE) tissue for screening *ROS1* or *KIF5B-RET* rearrangements; (4) complete survival data. The pathological diagnosis and staging of all tumors were re-evaluated by two expert pathologists according to the 2004 World Health Organization (WHO) classification, the tumor-node-metastasis staging system of the International Association for the Study of Lung Cancer (version 7), and the 2011 IASLC/ATS/ERS proposal [31]. For all patients, medical records were reviewed to extract data on clinicopathological characteristics. OS was measured from the date of diagnosis until the date of death or last follow-up (up until April 17, 2014). Patients lost to follow-up or deaths unrelated to NSCLC were omitted. This study has been approved by the institutional Research Medical Ethics Committee of Sun Yat-Sen University Cancer Center. All participants provided written informed consent for the genetic analysis.

### Tissue microarray construction

We collected tissues from 227 NSCLC patients with resected stage IIIA-N2 cancer, which were then formalin-fixed and paraffin-embedded. After hematoxylin and eosin staining, a representative tumor site was chosen for TMA which has been described in the literature [32]. Patients with ROS1- or RET-positive signals as revealed by the TMA panel were then confirmed with whole sections from the original tissue blocks.

### FISH

We established an ROS1 break-apart FISH assay using a break-apart probe (Vysis LSI ALK Dual Color, Abbott Molecular Inc., Des Plaines, IL) and a KIF5B-RET fusion FISH assay. The diagnostic criteria for the ROS1 FISH assay were in accordance with the ALK FISH assay [33,34].

*RET* rearrangement was performed with a KIF5B-RET fusion probe. Bacterial artificial chromosomes (BAC) clones were ordered from the Children's Hospital Oakland Research Institute (Oakland, CA). BAC DNA was extracted using Wizard Plus Midipreps DNA Purification System (Promega, Madison, WI). BAC clones RP11-167O6, RP11-460H18 (*KIF5B*) and RP11-351D16, RP11-124O11 (*RET*) were chosen to label the target genes. Probes were labeled with spectrum red and green (Vysis Inc., Downers Grove, IL) by BioPrime DNA labeling system (Invitrogen, Carlsbad, CA) [35].

FFPE slides were analyzed using an Olympus BX51 TRF microscope (Olympus, Tokyo, Japan) equipped with a triple-pass filter (DAPI/Green/Red). FISH results were scored by two independent experienced pathologists. Three major criteria for FISH diagnostic were as follows: (1) a minimum of 50 cells were evaluated; (2) ROS1 split signal was considered to be separated by at least two diameters in length; a fused signal was considered to be adjacent to or fused to a yellow signal; (3) at least 15% of the cells contained ROS1 split signals or KIF5B-RET fusion signals [36,37].

### Direct sequencing

All tumors with ROS1- or RET-positive signals, whether less than or more than 15%, were verified by direct sequencing. Specific PCR primers were summarized in S1 Table. PCR reactions were performed under the following conditions: 94°C for 30sec, 40 cycles with 94°C for 30sec, 58°C for 30sec, and 72°C for 1 min, with a final extension of 72°C for 5 min. Identified cases were tested at least twice.

### IHC

FFPE tissues were sectioned (4 μm) and stained using automated IHC for ROS1 expression in a Benchmark XT staining module (Ventana Medical Systems, Tucson, AZ). Then, the FFPE slides were deparaffinized, subjected to heat-mediated antigen retrieval and endogenous peroxidase inactivation. ROS1 antibody (rabbit monoclonal, clone D4D6, Cell Signaling Technology, Danvers, MA) was diluted to 1:50 and incubated at 37°C for 30 min. Signals were detected using an OptiView DAB IHC Detection Kit (Ventana Medical Systems, Tucson, AZ). The IHC ROS1-fusion positive control was confirmed by FISH, and the IHC ROS1-fusion negative control was confirmed by normal lung tissue.

### Statistical analysis

Statistical analysis consisted of Fisher’s exact test, the Chi-square test, and the Student’s t-test (comparison of means). The Kaplan-Meier method with a log-rank test was used to estimate OS. The Cox proportional hazards model was used to compare independent prognostic factors of biological and clinicopathological features. Two-sided statistical significance was defined as *p* < 0.05. All analyses were performed with SPSS 16.0 statistics software (SPSS Inc., Chicago, IL).

## Results

### Patient characteristics

We retrospectively enrolled 227 resected cases, and successfully detected 204 cases using a TMA panel. All tumors were previously treated with surgery and were diagnosed as stage IIIA-N2 cancer. Detailed clinicopathological characteristics are listed in Table 1.

**Table 1:** The relationship between clinicopathologic characteristics and *ROS1* rearrangement in 204 NSCLC patients.

Characteristics	Total (n, %)	*ROS1* rearrangement		*P* Value
		Negative	Positive	
**Number of patients (%)**	204	200 (98.0)	4 (2.0)	
**Age (years; %)**				
≤ 60	130 (63.7)	128 (98.5)	2 (1.5)	0.622
> 60	74 (36.3)	72 (97.3)	2 (2.7)	
**Gender (%)**				
male	143 (70.1)	139 (97.2)	4 (2.8)	0.319
female	61 (29.9)	61 (100.0)	0 (0.0)	
**Histology (%)**				
ADC	137 (67.2)	133 (97.1)	4 (2.9)	0.305
SCC	67 (32.8)	67 (100.0)	0 (0.0)	
**Differentiation (%)**				
well	3 (1.5)	3 (100.0)	0 (0.0)	1.000
moderate	84 (41.2)	82 (97.6)	2 (2.4)	
poor	117 (57.3)	115 (98.3)	2 (1.7)	
**T stage (%)**				
T1	7 (3.4)	7 (100.0)	0 (0.0)	0.411
T2	139 (68.1)	135 (97.1)	4 (2.9)	
T3	58 (28.5)	58 (100.0)	0 (0.0)	
**Smoking history (pack-years; %)**				
0	77 (37.7)	76 (98.7)	1 (1.3)	0.625
< 20	28 (13.7)	27 (96.4)	1 (3.6)	
≥ 20	99 (48.6)	97 (98.0)	2 (2.0)	
**ECOG (%)**				
≤ 1	194 (95.1)	190 (97.9)	4 (2.1)	1.000
>1	10 (4.9)	10 (100.0)	0 (0.0)	
**Surgical procedure (%)**				
pneumonectomy	44 (21.6)	44 (100.0)	0 (0.0)	0.579
lobectomy	160 (78.4)	156 (97.5)	4 (2.5)	
**Surgical resection (%)**				
R0	182 (89.2)	180 (98.9)	2 (1.1)	0.058
R1+R2	22 (10.8)	20 (90.9)	2 (9.1)	
**Tumor diameter (cm; %)**				
≤ 4.0	104 (51.0)	103 (99.0)	1 (1.0)	0.362
> 4.0	100 (49.0)	97 (97.0)	3 (3.0)	
**Tumor location (%)**				
peripheral	151 (74.0)	51 (96.2)	2 (3.8)	0.277
central	53 (26.0)	149 (98.7)	2 (1.3)	
**Postoperative chemotherapy (%)**				
Yes	127 (62.3)	123 (96.9)	4 (3.1)	0.299
No	77 (37.7)	77 (100.0)	0 (0.0)	
**Postoperative radiotherapy (%)**				
Yes	31 (15.2)	29 (93.5)	2 (6.5)	0.110
No	173 (84.8)	171 (98.8)	2 (1.2)	
**Pleural invasion (%)**				
Yes	123 (60.3)	121 (98.4)	2 (1.6)	0.650
No	81 (39.7)	79 (97.5)	2 (2.5)	

NSCLC, non-small cell lung cancer; ADC, adenocarcinoma; SCC, squamous cell carcinoma; ECOG, eastern cooperative oncology group; R, resection; ROS1, c-ros oncogene 1.

**Fig 1:**
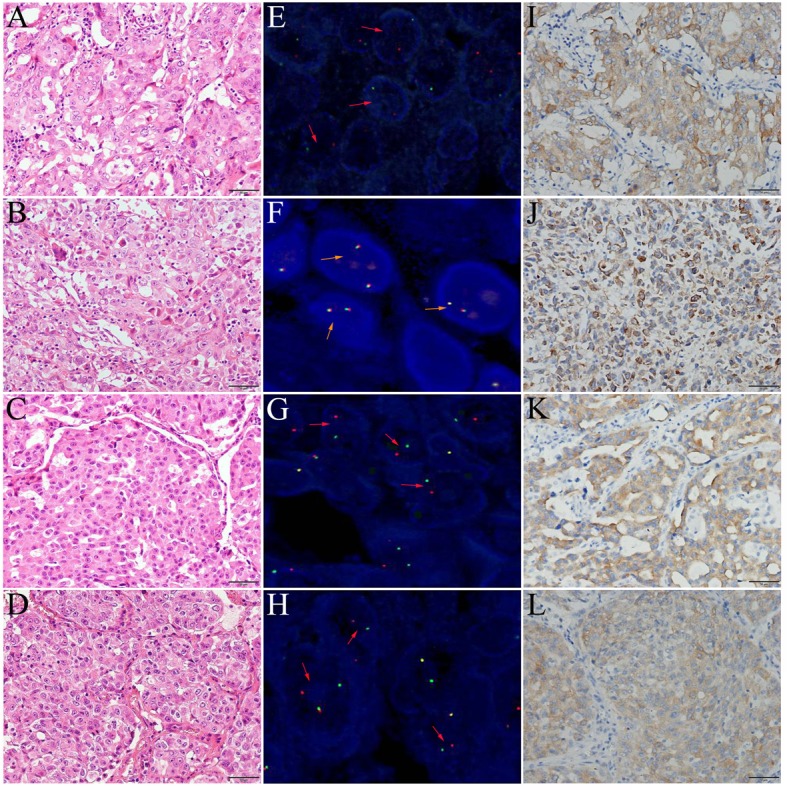
Diagnosis of 4 NSCLC cases with *ROS1* rearrangement in resected stage IIIA-N2. (A-D) It shows the light micrograph, revealing adenocarcinoma (HE 200 ×). (E-H) Red probes are hybridized to 5’ region of ROS1 and aqua probes to 3’ region of ROS1. Red arrows denote split signals in ROS1-fusion positive tumor cells. Orange arrows denote fusion signals in ROS1-fusion negative tumor cells. (I-L) Immunohistochemistry reveals the ALK expression in ROS1-fusion positive cases.

All 4 ROS1-rearranged cases detected by FISH were male and adenocarcinoma. The predominant pathological type of cancer was acinar pattern, except for one case containing a mixture of pathological types (acinar and mucous tumor cells) (Fig 1A–1D). Also, of the 204 NSCLC cases, there was no significant association of baseline characteristics between ROS1-fusion positive and negative groups (Table 1). The median age of ROS1-fusion positive cases tend to be younger than ROS1-fusion negative cases in the limited subsets (55 vs 58 years), although no significant difference was found (*p* = 0.867).

### Detected *ROS1* and *KIF5B-RET* rearrangements by two FISH assays

Using ROS1 FISH assay, 3 of 204 cases (1.5%) were diagnosed as ROS1 FISH-positive in the TMA panel (Fig 1E, 1G and 1H). In 137 adenocarcinoma cases, the frequency was 2.2% (3/137) by FISH assay. Clinicopathologic characteristics of ROS1 FISH-positive cases were listed in Table 2. In addition, we observed 7 ROS1 FISH-negative cases with less than 15% FISH-positive signals (data not shown).

**Table 2:** Summary of 4 NSCLC patients with *ROS1* rearrangement analyzed by FISH, direct sequencing and IHC.

Patients NO.	Age (years)	Gender	Smoking History (pack-years)	Histology	Differentiation	FISH (%)	Direct Sequencing	IHC	OS (months)	Survival Status
**1**	45	M	20	acinar	moderate	19	SDC4:ROS1 (E2/E32)	positive	7.43	Dead
**2**	47	M	15	acinar	poor	10	SDC4:ROS1 (E4/E32)	positive	39.30	Dead
**3**	62	M	0	acinar	poor	20	SDC4:ROS1 (E4/E34)	positive	27.03	Dead
**4**	69	M	40	acinar+mucous	moderate	18	negative	positive	11.27	Dead

NSCLC, non-small cell lung cancer; ROS1, c-ros oncogene 1; FISH, fluorescence in situ hybridization; IHC, immunohistochemistry; M, male; OS, overall survival.

Using KIF5B-RET FISH assay, we found no RET FISH positive cases. And 6 tumors were also found KIF5B-RET fusion signals, however, less than 15% according to the criteria (data not shown).

### Detected *ROS1* and *KIF5B-RET* rearrangements by direct sequencing

**Fig 2:**
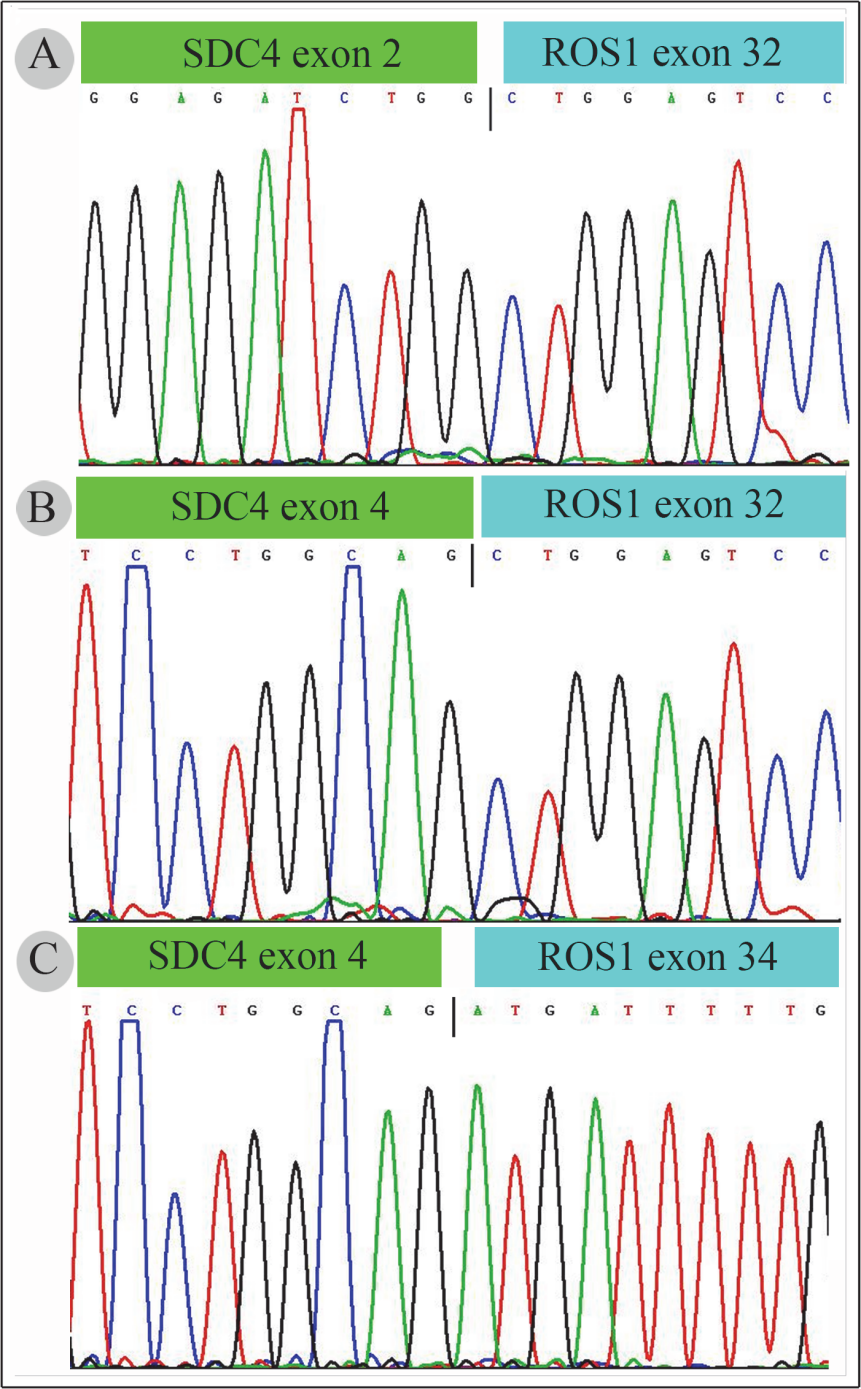
The result of direct sequencing in ROS1-rearranged NSCLC. (A) SDC4:ROS1 (E2/E32). (B) SDC4:ROS1 (E4/E32). (C) SDC4:ROS1 (E4/E34).

Tumors containing ROS1- or RET-positive signals, whether less than or more than 15% signal, were all confirmed by direct sequencing, and 3 cases (1.5%) were verified with *ROS1* rearrangement (Table 2). Two cases were found to have *ROS1* rearrangement, which was consistent with the ROS1 FISH assay. However, 1 ROS1 FISH-positive case was confirmed as negative by direct sequencing. When verifying the 7 ROS1 FISH-negative cases with less than 15% fusion signals, 1 case was found to have *ROS1* rearrangement (Fig 1F), but no *KIF5B-RET* rearrangement case was found. Variants of *ROS1* rearrangement were SDC4-ROS1 (E2:E32), SDC4-ROS1 (E4:E32) and SDC4-ROS1 (E4:E34), respectively (Fig 2). Detailed clinicopathologic characteristics were summarized in Table 2.

### Confirmed *ROS1* rearrangement by IHC

IHC was further used to confirm the 4 ROS1-fusion positive cases detected by FISH or direct sequencing and all were IHC positive. The extent of ROS1-rearranged cases was more than 75% and intensities were 2+ and 3+ (Fig 1I–1L). IHC revealed 3+ intensity for two ROS1-rearranged cases detected by FISH and direct sequencing (Fig 1I and 1K), a 2+ intensity for one ROS1 FISH-positive case confirmed as negative by direct sequencing (Fig 1L), and 3+ intensity for one ROS1 FISH-negative case confirmed as positive by direct sequencing (Fig 1J).

### Survival analysis

**Fig 3:**
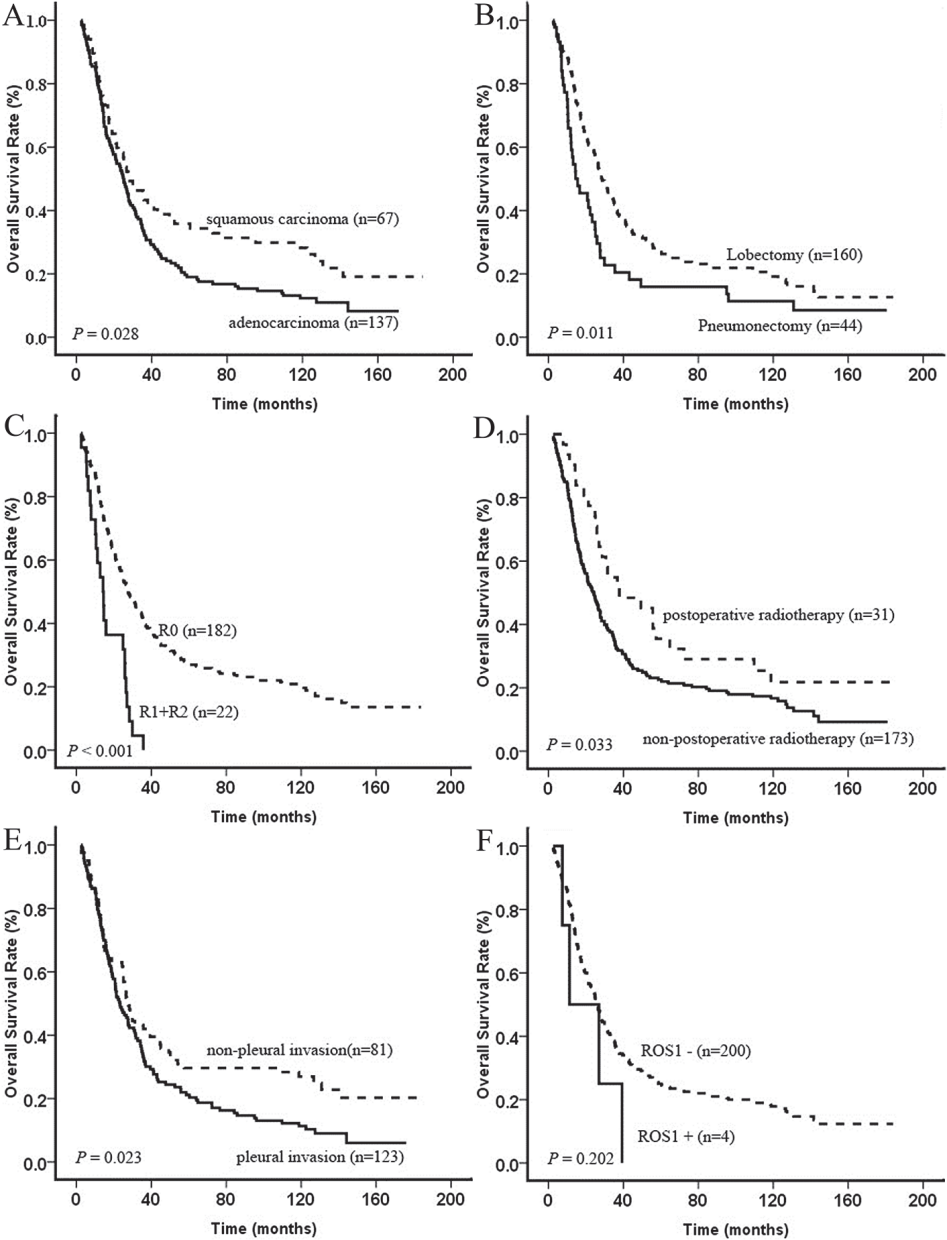
Comparison of overall survival in Kaplan-Meier survival curve analysis. Overall survival curves stratified by (A) histology status (B) surgical procedure, (C) surgical resection, (D) postoperative radiotherapy, (E) pleural invasion, and (F) ROS1 status.

Out of 204 cases available for survival analysis, 174 (85.3%) death events occurred during the follow-up period. The median overall survival time was 25.9 months (range 2.57 to 184 months). The 5-year survival rate was 14.7%. There were significant associations with histology (*p* = 0.028), surgical procedure (*p* = 0.011), surgical resection (*p* < 0.001), postoperative radiotherapy (*p* = 0.033), and pleural invasion (*p* = 0.023) (Fig 3A–3E). No significant association was found in other clinicopathological profiles (Table 3). Multivariate analysis for OS suggested prognostic factors as depicted in Table 4. The median overall survival time in ROS1-fusion positive group was shorter than that in ROS1-fusion negative group (19.15 vs 25.92 months).

**Table 3:** Log-Rank test analysis of OS in 204 NSCLC patients.

Characteristics	Subset	*P* Value
		OS
**Age (years)**	≤ 60 *vs* > 60	0.425
**Gender**	male *vs* female	0.863
**Histology**	ADC *vs* SCC	0.028
**Differentiation**	well *vs* moderate *vs* poor	0.932
**T stage**	T1 *vs* T2 *vs* T3	0.323
**Smoking history (pack-years)**	0 *vs* < 20 *vs* ≥ 20	0.503
**ECOG**	≤ 1 *vs* >1	0.836
**Surgical procedure**	pneumonectomy *vs* lobectomy	0.011
**Surgical resection**	R0 *vs* R1+R2	< 0.001
**tumor diameter (cm)**	≤ 4.0 *vs* > 4.0	0.818
**tumor location**	central *vs* peripheral	0.701
**Postoperative chemotherapy**	yes *vs* no	0.199
**Postoperative radiotherapy**	yes *vs* no	0.033
**Pleural invasion**	yes *vs* no	0.023
**ROS1 rearragement**	negative *vs* positive	0.202

OS, overall survival; DFS, disease-free survival; NSCLC, non-small cell lung cancer; ADC, adenocarcinoma; SCC, squamous cell carcinoma; ECOG, eastern cooperative oncology group; R, resection; ROS1, c-ros oncogene 1.

**Table 4:** Multivariate analysis of OS in 204 NSCLC patients.

Variable	Subset	OS	
		HR (95% CI)	*P* Value
**Histology**	ADC *vs* SCC	0.669 (0.462–0.968)	0.033
**Surgical procedure**	pneumonectomy *vs* lobectomy	0.549 (0.370–0.814)	0.003
**Surgical resection**	R0 *vs* R1+R2	3.497 (2.107–5.803)	< 0.001
**Pleural invasion**	yes *vs* no	1.566 (1.099–2.233)	0.013

OS, overall survival; NSCLC, non-small cell lung cancer; HR, hazard ratio; CI, confidence interval; ADC, adenocarcinoma; SCC, squamous cell carcinoma; R, resection.

## Discussion

The prognosis of resected stage IIIA-N2 NSCLC is relatively poor, for which optimal treatment remains controversial. Target therapy, an effective treatment strategy based on target genes, is promising to improve the predicament. The aim of this study was to determine the frequency of *ROS1* and *RET* rearrangements and their relationship with clinicopathological characteristics in resected stage IIIA-N2 NSCLC and to provide guidance for future clinical treatment. *ROS1* and *RET* rearrangements appear to occur in approximately 2% of NSCLC [22–24,38,39]. By FISH assay, we screened 204 cases and identified approximately 1.5% (3/204) NSCLC cases harboring *ROS1* rearrangement, but found no *KIF5B-RET* rearrangement. By direct sequencing, 3 cases (1.5%) were verified to harbor *ROS1* rearrangement, among tumors with more than or less than 15% fusion positive signals. Using IHC assay, 4 ROS1-fusion positive cases were determined with *ROS1* rearrangement, including two inconsistent cases that one ROS1 FISH-positive case was confirmed as negative and another ROS1 FISH-negative case was found *ROS1* rearrangement by direct sequencing. In total, 4 cases (2.0%) were identified to have *ROS1* rearrangement confirmed by different methods. The frequency indicated a low frequency of this rearrangement in NSCLC, which was consistent with the previous reports [7,13,14,40–42].

Precise diagnosis is the basis of choosing an appropriate therapy. Detecting by FISH assay, Bergethon’s group reported 1.7% of NSCLC patients (18/1073) had *ROS1* rearrangement [7]. Takeuchi and colleagues reported 0.9% of NSCLC cases (13/1476) with *ROS1* rearrangement [24]. In addition, Li and his colleagues showed that 1% of NSCLC cases (2/202) had *ROS1* rearrangement detected by RT-PCR [43]. With quantitative real-time reverse transcriptase PCR (qRT-PCR) and RT-PCR, Yan’s group observed an ROS1-rearranged incidence of 1% (11 of 1139 cases) and with IHC, FISH and RT-PCR, Rimkunas and his co-workers reported that 1.6% (9 of 556 cases) of NSCLC cases were *ROS1* rearrangement [22,39]. In this study, 2% of resected stage IIIA-N2 NSCLC cases had *ROS1* rearrangement. It indicated that the frequency of *ROS1* rearrangement in current study was consistent with the previous researches and no much difference was found between resected stage IIIA-N2 NSCLC and all stage NSCLC.

The patient selection is also an important issue for targeted therapy, especially for locally advanced NSCLC. Previous studies showed that the clinical characteristics of ROS1-fusion positive patients were the young, the nonsmoker, and the adenocarcinoma patients who might benefit most from targeted therapy [13,44]. In our study, a total of 4 cases with *ROS1* rearrangement were all adenocarcinomas. So we analyzed the 137 adenocarcinoma cases in this cohort and found that the enrichment of adenocarcinomas yielded greater frequency (2.9%) of *ROS1* rearrangement compared with all NSCLC cases reported by previous studies [7,22,24]. The median age of ROS1-fusion positive patients was younger than ROS1-fusion negative cases in the limited subsets (55 vs 58 years), suggesting that *ROS1* rearrangement in resected stage IIIA-N2 NSCLC occurred mainly in certain subgroups, such as the young and those with adenocarcinoma. Seventy-five percent of ROS1-fusion positive cases were smokers, which differed from previous reports. Several possible reasons may explain this discrepancy. First, a selective bias may exist. Second, there are 52.9% male smokers and 2.4% female smokers in China and Chinese men who smoke outnumber male smokers in developed countries [45]. No *ROS1* rearrangement was identified with squamous cell carcinoma in our cohort. However, Davies’s group reported 2 of 428 cases with *ROS1* rearrangement in squamous carcinoma [18]. In squamous cell carcinoma cases, genetic abnormalities are rare, and the therapy response of ROS1-fusion positive cases with squamous cell carcinoma still need further study.

In ROS1-rearranged cases, the predominant histology subtypes were mucinous cribriform pattern or signet ring cells in previous reports [46,47]. Other research with a small series of 15 surgical ALK-rearranged cases described solid growth with signet-ring cells or cribriform architecture with abundant extracellular mucus in more than half of ROS1-fusion positive cases [47]. However, the main histological type identified in our research was the acinar pattern, except for one case as previously described. It indicated that in resected stage IIIA-N2 NSCLC cases, patients with acinar pattern could have higher frequency of *ROS* rearrangement, and the histological characteristic would help to select patients who will benefit from the targeted inhibitors.

The prognosis of resected stage IIIA-N2 NSCLC is unsatisfactory, but the higher frequency of *ROS1* rearrangement in stage IIIA-N2 adenocarcinoma cases reminds that targeted therapy will be a new treatment strategy. *ROS1* gene shares a high degree of amino acid homology with *ALK* gene, specifically within the ATP-binding site [48]. A recent report from a phase I trial described early evidence of clinical response to crizotinib in ROS1-rearranged advanced NSCLC patients and that the preliminary response rate and disease-control rate were 57% and 79%, respectively [7,49]. Other clinical trials with second-generation ALK inhibitors enrolled patients harboring *ROS1* rearrangement such as AP26113 (NCT01449461) and ASP3026 (NCT01284192) these are ongoing [49].

Analysis indicated that no specific clinicopathological feature was significantly associated with the ROS1-rearranged status in resected stage IIIA-N2 NSCLC. Cai’s group reported that ROS1-fusion negative patients had greater OS than ROS1-fusion positive patients [31]. Similarly, our study showed that the median OS in ROS1-fusion positive patients was shorter than ROS1-fusion negative patients, although not reaching statistical significance due to limited study sample (19.15 vs 25.92 months; *p* = 0.202) (Fig 3F). Multivariate analysis for OS indicated that squamous cell carcinoma and lobectomy were independent predictors of poor prognosis; R0 surgical resection and non-pleural invasion were independent predictors of good prognosis. Takeuchi’s group found that four independent factors, such as being older than 50 years-of-age, male, high pathological stage and negative kinase-fusion status were indicators of poor prognosis [24]. Multivariate analysis by Lee and colleagues showed that ROS1 expression was an independent factor for poor prognosis in cases with stage I adenocarcinoma [50]. However, the low frequency of *ROS1* rearrangement and limited study samples precluded us from further evaluating the significance of *ROS1* rearrangement in this paper.

We do aware that several limitations of this study should also be considered. Our study was a relatively small retrospective cohort of resected stage IIIA-N2 NSCLC patients, and all tumors were selected from one institution. The number of ROS1-rearranged cases was small as well. In addition, testing *ROS1* and *RET* rearrangements with a TMA panel may increase the incidence of false-negative. Only tumors with break-apart signals were confirmed by direct sequencing and IHC, because many specimens are insufficient. Further prospective clinical trials with a relatively large study sample should be conducted to evaluate the effect of target therapy in resected stage IIIA-N2 NSCLC.

In conclusion, 2% of 204 cases had been detected with *ROS1* rearrangement. The identification of 4 ROS1-rearranged cases supports the concept that the FISH assay is an optimal method for detecting *ROS1* rearrangement in clinical application, but additional validation methods could also be used if it is necessary. In resected stage IIIA-N2 NSCLC, ROS1-rearranged cases tended to occur in younger patients with adenocarcinomas. The prognosis of resected stage IIIA-N2 NSCLC is generally considered poor, but ROS1-rearranged patients may benefit from the target therapy and prolong their survival.

## Supporting Information

S1 Table The special primer sequences of direct sequencing for *ROS1* rearrangement. (XLS)
